# Supplementary material for: Candidate genes for stem rust resistance in Italian ryegrass revealed by nested association mapping
Source: Theor Appl Genet. 2026 Jul 28;139(8):218. doi: 10.1007/s00122-026-05321-7 (PMC13415492; doi:10.1007/s00122-026-05321-7)
Supplement: Supplementary file 1 — Supplementary file1 (DOCX 612 KB) [file 122_2026_5321_MOESM1_ESM.docx]

# Supplementary files

**Supplementary Table 1:** Identification of single nucleotide polymorphisms (SNPs) associated with stem rust resistance that have a *p*-value smaller than *p* = 1.0 × 10^-6^ but are below the Bonferroni significance threshold of 5% in a nested association mapping population (NAM) of Italian ryegrass (*Lolium multiflorum* Lam.). The NAM population consists of 708 F_2_ individuals derived from 24 founder plants exhibiting high variation in stem rust resistance. As phenotypic data, the best unbiased linear estimators from three different environments were used. The genotypic matrix consists of 3,199,253 SNP markers. Gene name represents the name of the gene in the reference genome cv. `Rabiosa` (Chen et al. 2025). The gene function was revealed by basic local alignment search tool. The allelic effect means the effect of alternative allele.

| Chromosome | SNP position (bp) | Allelic effect | Phenotypic variance (%) | Gene position (bp) | Gene name | SNP effect |
| --- | --- | --- | --- | --- | --- | --- |
| 1 | 165,319,368 | 1.26 | 4.00 | - | *-* |  |
| 6 | 118,700,714 | 1.76 | 3.90 | 118696039-118704239 | *Chr6.15818* | Synonymous variant, intron variant |


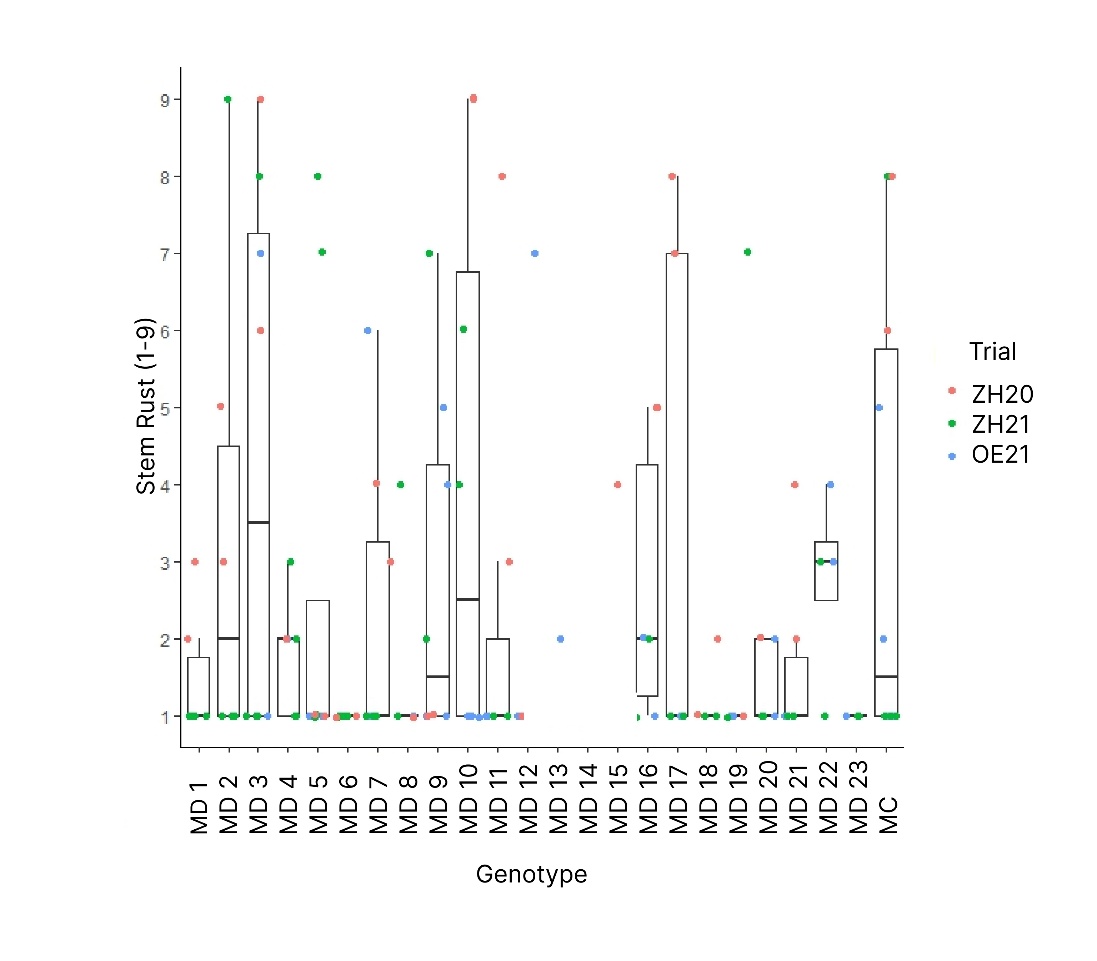


**Supplementary Fig. 1** Stem rust infection scores of the founder plants across three independent field trials. Stem rust was scored on a scale from 1 (no stem rust symptoms) to 9 (plants fully covered by stem rust). The x-axis shows the founder plant genotypes, and the y-axis shows the raw stem rust infection scores. Data are presented separately for each trial to illustrate the variation in disease response under natural infection conditions.


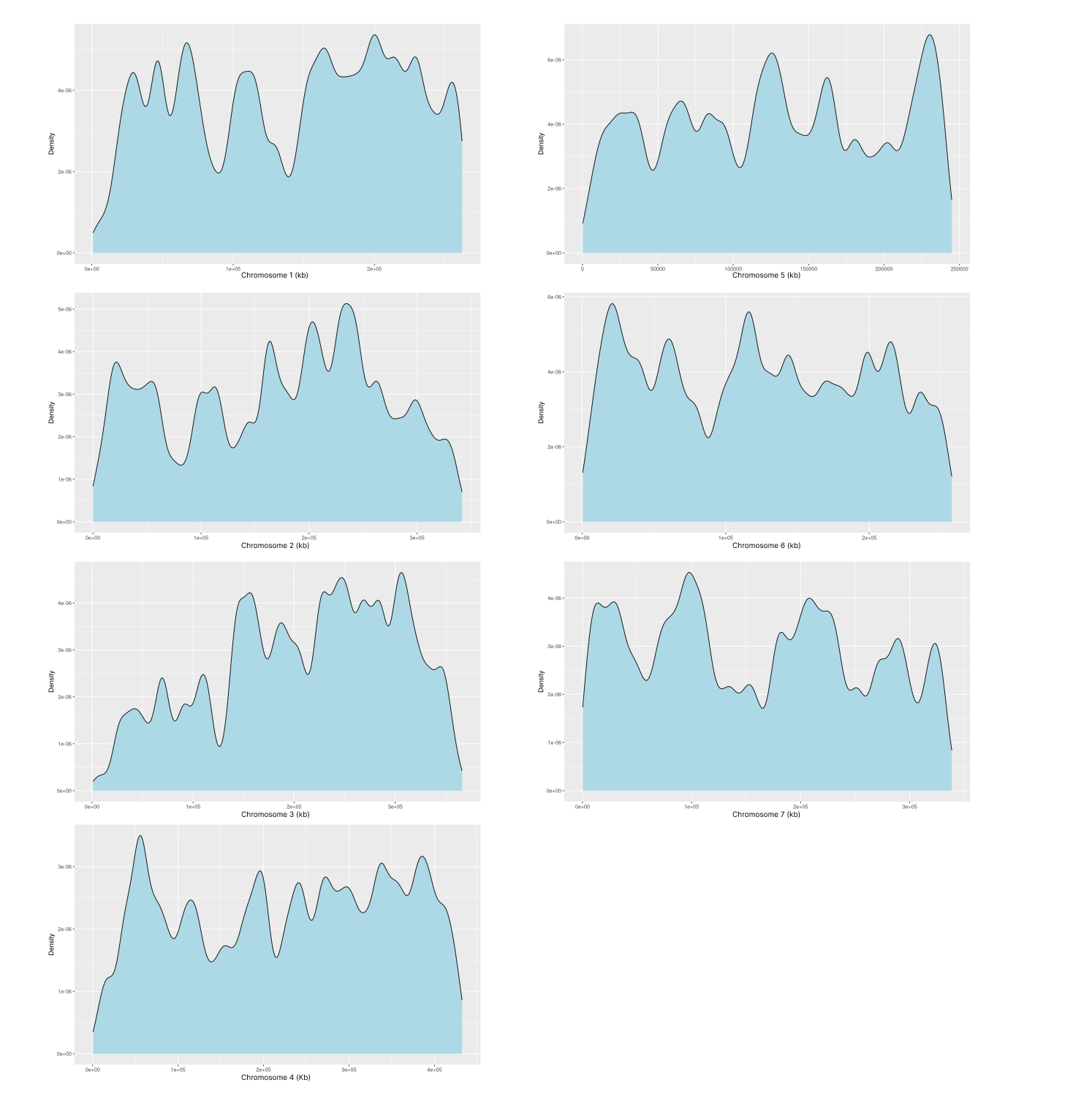


**Supplementary Fig. 2** Density of single nucleotide polymorphism (SNP) markers along the seven chromosomes of reference genome cv. `Rabiosa` (Chen et al. 2025). Marker density is shown for each chromosome separately. The x-axes show the length of each chromosome in kb and the y axes display the density of the markers.


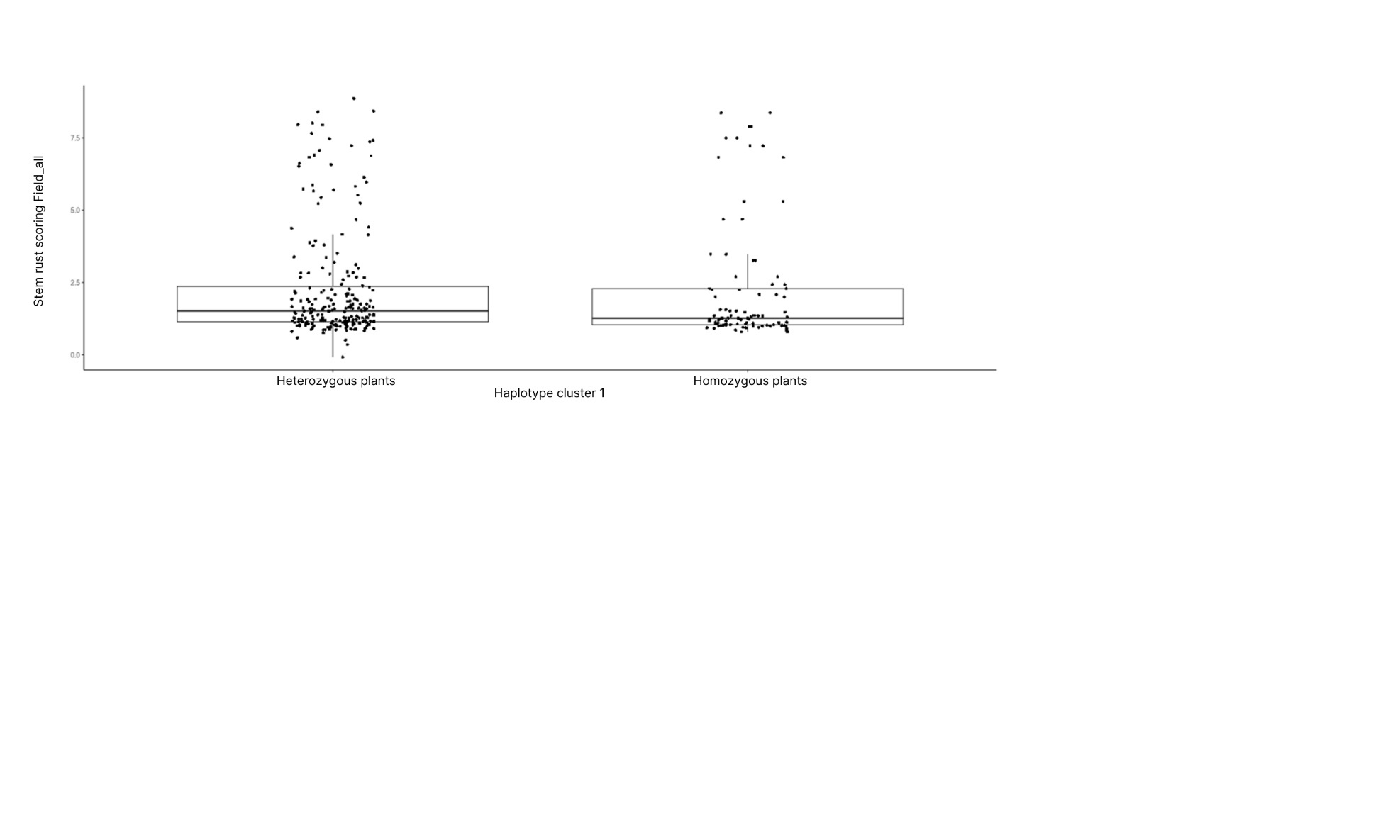


**Supplementary Fig. 3** Haplotype cluster 1 divided into two groups. If a plant has two copies of haplotype cluster1, it is considered homozygous. If the plant has only one allele of haplotype cluster 1 and the second one belongs to one of the other haplotype clusters, it is labeled heterozygous plant. The y-axis displays the stem rust scoring for all field data using the best linear unbiased estimator. Homozygous plants showed significantly lower stem rust symptoms than heterozygous plants (p-value: < 2.2e-16).
